# Attitudes Towards Digital Health Interventions in Germany: Findings From a Population-Based Representative Survey

**DOI:** 10.32872/cpe.15233

**Published:** 2025-08-29

**Authors:** Lena Sophia Steubl, Rebekka Büscher, Lasse Bosse Sander, Amit Baumel, Katja Barck, Cedric Sachser, Jörg Michael Fegert, Elmar Brähler, Harald Baumeister, Matthias Domhardt

**Affiliations:** 1 Department of Clinical Psychology and Psychotherapy, Institute of Psychology and Education, Ulm University, Ulm, Germany; 2 Medical Psychology and Medical Sociology, Medical Faculty, University of Freiburg, Freiburg im Breisgau, Germany; 3 Department of Clinical, Neuro and Developmental Psychology, Vrije Universiteit Amsterdam, Amsterdam, The Netherlands; 4 Department of Community Mental Health, University of Haifa, Haifa, Israel; 5 Department of Child and Adolescent Psychiatry/Psychotherapy, Medical Faculty, Ulm University, Ulm, Germany; 6 Department of Psychosomatic Medicine and Psychotherapy, University Medical Center Mainz, Mainz, Germany; 7 Medical Psychology and Medical Sociology, Medical Faculty, University of Leipzig, Leipzig, Germany; Friedrich-Alexander-Universität Erlangen-Nürnberg, Erlangen, Germany

**Keywords:** e-health, acceptance, UTAUT, digital psychotherapy, online therapy

## Abstract

**Background:**

Digital (mental) health interventions have the potential to address barriers in mental health care. However, attitudes towards these interventions are a crucial factor to their successful implementation. Therefore, this study aims to assess those in a representative sample of the German adult population.

**Method:**

A total of *N* = 2,519 participants took part in the survey as part of a larger study. Following a structured face-to-face interview, participants completed a self-administered questionnaire under the supervision of the interviewer. The questionnaire was based on the E-Therapy Attitudes Measure (ETAM) and the Attitudes towards Psychological Interventions Questionnaire (APOI). Results were analyzed by means of Pearson's product moment correlation coefficients and Spearman's ρ statistics. Supplementary open-ended questions explored participants' utilization of digital health interventions for specific conditions, the conditions they perceived as suitable for those, and the perceived barriers to their adoption. Replies on open-ended questions are summarized descriptively.

**Results:**

While a majority of participants (34.0%–41.5%) indicated partial agreement with the potential usefulness and advantages of digital health interventions (Items 1-3), a substantial proportion (45.8%, 95% CI [43.8%, 47.7%]) expressed an entire refusal to use them for future psychological problems (Item 4). Older individuals and those with lower educational status expressed particular critical views. Key barriers identified by participants comprised the absence of personal contact, technical issues, and concerns related to data privacy and security.

**Conclusion:**

The results of this study indicate that while participants acknowledge the potential benefits of digital health interventions, the observed limited acceptance rates and identified barriers are to be addressed, in order to fully harness their potential.

## Highlights

- A substantial proportion expressed an absolute reluctance to use digital health interventions.
- This reservedness decreases with younger age and higher educational status.
- Key barriers include the absence of personal contact, technical issues, and data privacy and security concerns.
- There was, however, a subsample that would rather use digital than regular face-to-face treatment.

While the precise prevalence rates of common mental conditions differ across countries, they are consistently high worldwide – with female gender, younger age, living without a partner, and low socio-economic status representing important risk factors (Baumeister & Härter, 2007; GBD 2019 Mental Disorders Collaborators, 2022; Jacobi et al., 2014; World Health Organization, 2022). Mental conditions are associated with substantial individual and societal costs and efficacious interventions to treat them are theoretically present, but only a fraction of affected individuals receive an evidence-based treatment (Mack et al., 2014; Wang et al., 2007). Possible reasons for the low uptake rates of mental health care include logistical barriers, fear of stigmatization, and a shortage of actually available treatment options (Brakemeier & Herpertz, 2019; Hansen et al., 2002; Lambert, 2017).

Expanding the provision of digital health interventions has repeatedly been linked to the promise to overcome these barriers (Andersson & Titov, 2014; Domhardt, Cuijpers, et al., 2021; Torous et al., 2021). Digital health interventions, defined as interventions using the Internet as a medium for the delivery of psychotherapeutic treatment, have been found to be efficacious and effective for a variety of mental conditions in a variety of populations (Domhardt et al., 2019, 2020; Domhardt, Schröder, et al., 2021; Edge et al., 2023; Kolaas et al., 2024; Lecomte et al., 2020; Moshe et al., 2021; Plessen et al., 2025; Steubl et al., 2021). These interventions make therapeutic content available digitally – with the help of for example audios, videos, texts, downloads, and questions. A recent framework provides information on how to implement and study them (Smith et al., 2023). Of note, while video-based psychotherapy (i.e., synchronous therapy via videoconferencing) is also Internet-delivered, it differs from the types of digital interventions primarily referred to in this study and is therefore not the main focus of the present analysis.

Compared to conventional face-to-face treatments, digital health interventions may be easier to access, integration into the patients’ daily lives may be more flexible and the provision may be easily scalable and cost-efficient (at least compared to non-bonafide comparisons) once developed (Andersson & Titov, 2014; Gega et al., 2022; Kählke et al., 2022; Titov et al., 2015; Warmerdam et al., 2010). However, there are two issues that have been repeatedly associated with limited implementation success of digital health interventions into routine health care: low uptake and low adherence (Kählke et al., 2022; Lambert, 2017).

One suggested reason for these issues may be the possible low level of patients’ acceptance of digital health interventions. This is in line with the basic assumption of the so-called Unified Theory of Acceptance and Use of Technology (UTAUT), an overarching framework that combines eight models and postulates that acceptance is a direct predictor of the future use of digital interventions (Venkatesh et al., 2003). Along with several moderators (e.g., gender, age, voluntariness), UTAUT hypothesizes four determinants of intention to use as a predictor of behavior (i.e., usage): 1) the degree to which an individual believes that the technology will help them (*Performance Expectancy*), 2) the expected effort of using the technology (*Effort Expectancy*), 3) the influence of the opinion of significant others (i.e., peers or others, *Social Influence*), and 4) the degree to which necessary resources and support are seemingly available (*Facilitating Conditions;* Venkatesh et al., 2003). The UTAUT concept has been widely and successfully used in multiple areas, including digital health (Dwivedi et al., 2020).

A scoping review found evidence that digital health interventions were perceived as less beneficial compared to traditional face-to-face interventions (Apolinário-Hagen, Kemper, et al., 2017). Furthermore, the intentions to utilize digital health interventions in the future were generally lower in comparison to face-to-face services. Damerau et al. (2021) assessed the acceptance of digital health interventions following the UTAUT model in patients with diabetes in Germany, resulting in moderate acceptance ratings. Hereby, gender and having a mental disorder had a significant influence on acceptance, with participants with mental disorders having a significantly higher acceptance than those without. More broadly, Philippi et al. (2021) conducted secondary analyses on primary data of ten studies from Germany that examined participants' acceptance of digital health interventions using the UTAUT framework. It was found that the most appropriate model resembled the fundamental structure of UTAUT, with performance expectancy emerging as the most influential predictor (Philippi et al., 2021). On average, the level of acceptance observed was categorized as low to moderate, primarily being drawn from a segment of the population that was close to hand (i.e., convenience sampling; Philippi et al., 2021). All included studies focused on specific disorders or populations (i.e., depression, diabetes and comorbid depression, chronic pain, well-being and health in the elderly, gastrointestinal problems, aftercare for inpatients, multiple sclerosis, psychotherapists’ acceptance towards blended therapy; Philippi et al., 2021). However, another recent study on the acceptance of digital health interventions for depression care in Germany focusing on the perspective of patients, their relatives, and health professionals resulted in general openness towards the use of those interventions (Hafner et al., 2022). More precisely, about 80% of the participants reported being open to using a digital health intervention for depression (Hafner et al., 2022). Similar results have been found in two survey studies in Germany (Apolinário-Hagen et al., 2018; Apolinário-Hagen, Vehreschild, et al., 2017) concluding that the majority of participants regard digital health interventions as potentially helpful. Nevertheless, intentions to use are low and face-to-face treatments are preferred overall (Apolinário-Hagen et al., 2018; Apolinário-Hagen, Vehreschild, et al., 2017).

While these results shed some light on the attitudes towards digital health interventions – in the German population and in other western countries – they are still limited and inconclusive regarding attitudes towards digital health interventions in specific population groups, where acceptance levels are particularly high or low, and how this correlates with sociodemographic characteristics. Not only do the rates of acceptance diverge, there are also important limitations when it comes to the recruitment of survey participants. In particular, even recruitment for studies focusing on the general population was often convenience- and web-based and took place through digital advertisements (Apolinário-Hagen et al., 2018; Apolinário-Hagen, Vehreschild, et al., 2017) potentially leading to biased findings of prior research. This may restrict the generalizability of these findings to the general population. Therefore, this study aims to assess general attitudes towards digital health interventions exemplified in the German population by means of a representative and randomly chosen sample, in order to inform future efforts to integrate those interventions into routine health care.

## Method

The present study analyzes relevant parts of the data from a large, representative survey conducted in Germany. The survey was approved by the Ethics Committee of the Medical Faculty of the University of Leipzig (approval number: 298/21‐ek). The implementation of the survey and data collection was performed by an independent institute (USUMA, Berlin) between December 2020 and March 2021. To put the data collection period into perspective, it is important to note that the first DiGA (i.e., a certified health application that can be reimbursed by German health insurances) was available in October 2020 (Ludewig et al., 2021). Regional areas were first predefined using the ADM-Sampling-System (Heckel & Hofmann, 2014). Next, target households were selected by means of a random route procedure. For multi-person households, one person was randomly selected. This multi-stage recruitment strategy ensures representativity of included individuals (Heckel & Hofmann, 2014). No financial compensation or other incentives were provided to participants. All randomly selected participants were first informed verbally about the research background of the study as well as the voluntary nature and the right of later withdrawal of their own participation. Experienced and trained interviewers then conducted face-to-face interviews with 2,519 participants representative for the German population over the age of 15 and supervised also the accomplishment of the self-report questionnaires after participants gave their informed consent. The data predominantly relevant for this study was obtained in the self-report questionnaires.

### Measures

Relevant sociodemographic information (i.e., age, gender, marital status, level of education, religious affiliation, current stress, prior experience with digital health interventions) was collected from all participants.

Attitudes towards digital health interventions were assessed using four items (Items 1-4; 5-point Likert scale; 1 = absolutely no, 2 = rather no, 3 = partly, 4 = rather yes, 5 = absolutely yes) that were based on the “E-Therapy Attitude Measure” (ETAM; Apolinário-Hagen, Vehreschild, et al., 2017) and the “Attitudes towards Psychological Interventions Questionnaire” (APOI; Schröder et al., 2015) following the UTAUT model. Additionally, they were asked if they would use routine face-to-face psychotherapy for psychological problems in the future (5-point Likert scale, Item 5). Furthermore, three open questions were included in order to complement the quantitative measures and provide further exploratory insights. They assessed (1) the conditions for which participants already use digital health interventions, (2) the conditions participants could imagine to use digital health interventions in the future, and (3) possible barriers for the personal usage of digital health interventions. All questions were administered in German. To make sure patients understood what we conceive as digital health interventions, a short explanation was prefaced stating that the interventions in question are administered via the Internet (e.g., PC, tablet, smartphone) and can be used for the treatment of mental disorders, such as anxiety and depression, but also for other (mental and somatic) conditions. A translation of the full questionnaire, including the initial instruction, can be found in the Supplementary Materials (see Steubl et al., 2025S).

### Statistical Analyses

Statistical analyses were performed using R Studio (version 4.2.2; R Core Team, 2022). To classify attitudes towards digital health interventions descriptive analyses were used. Potential predictors (i.e., age, gender, highest level of education, current stress) were explored using Pearson's product moment correlation coefficients and Spearman's ρ statistics. Hereby, the four questions assessing attitudes towards digital health interventions were combined to one sum score. Results from open questions were qualitatively analyzed using a structured approach informed by conventional content analysis. One rater developed a coding scheme based on the content of the responses (LSS), which was then independently applied by a second rater (AF). Disagreements were resolved through discussion until consensus was achieved.

## Results

Of the 2,519 study participants, 1,322 participants self-rated their gender as female (52.5%), 1,130 as male (47.4%), and four as diverse (0.2%). The mean age was 50.3 years (*SD* = 18.1). Further characteristics of the study sample are described in Table 1.

**Table 1:** Characteristics of the Study Sample

Variable	*n* (%) or *M* (*SD*)
**Age (*M*, *SD*)**	50.3 (18.1)
Gender (*n*, %)	
Male	1,193 (47.4)
Female	1,322 (52.5)
Diverse	4 (0.2)
Marital status (*n*, %)	
Married, living together	1,076 (42.7)
Married, living apart	65 (2.6)
Single	757 (30.1)
Divorced	370 (14.7)
Widowed	243 (9.7)
German citizenship (*n*, %)	2,425 (96.3)
Highest level of education (*n*, %)	
In school	47 (1.9)
No graduation	57 (2.3)
Year 9 lower secondary school certificate)	679 (27.0)
Year 10 lower secondary school certificate	822 (32.7)
Graduated from polytechnical high school	240 (9.6)
Graduated from technical college with no accreditation	101 (4.0)
Entrance qualification for technical college/university	277 (11.0)
College/university studies completed	274 (10.9)
Other	3 (0.1)
Currently stressed (*n*, %)	
Absolutely no	1,388 (55.1)
Rather no	507 (20.1)
Partly	364 (14.5)
Rather yes	189 (7.5)
Absolutely yes	51 (2.0)
Previous usage of digital health interventions (*n*, %)	
Yes	17 (0.7)
No	2,497 (99.1)
Not stated	5 (0.2)
Heard of digital health interventions (*n*, %)	
Yes	873 (34.7)
No	1,593 (63.2)
Not stated	53 (2.1)

### Attitudes Towards Digital Health Interventions

**Figure 1:**
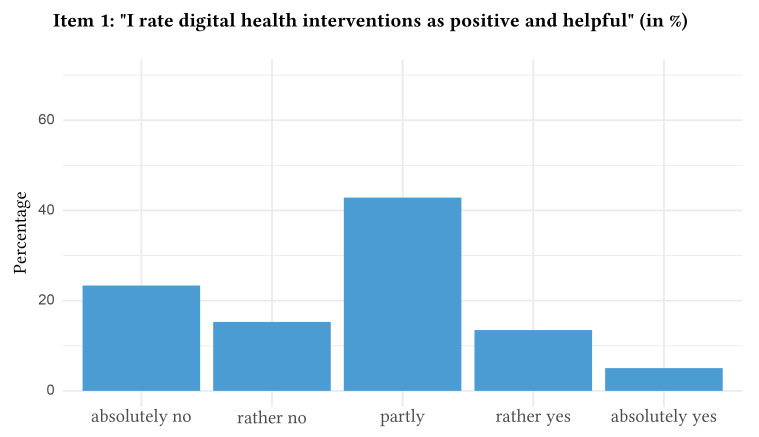
Attitudes Towards Digital Health Interventions: Item 1

**Figure 2:**
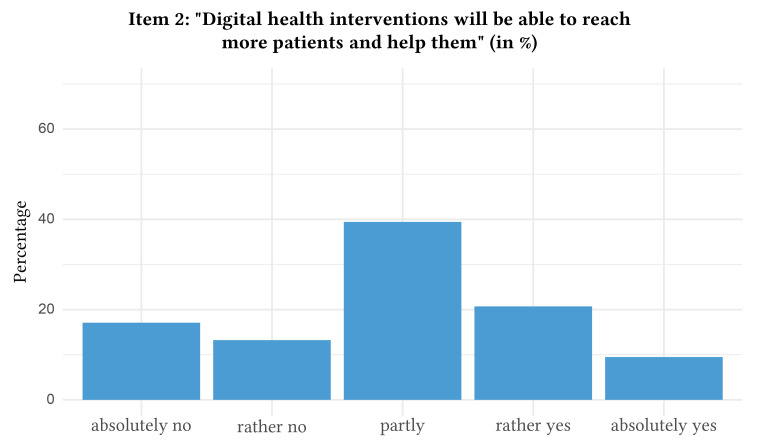
Attitudes Towards Digital Health Interventions: Item 2

**Figure 3:**
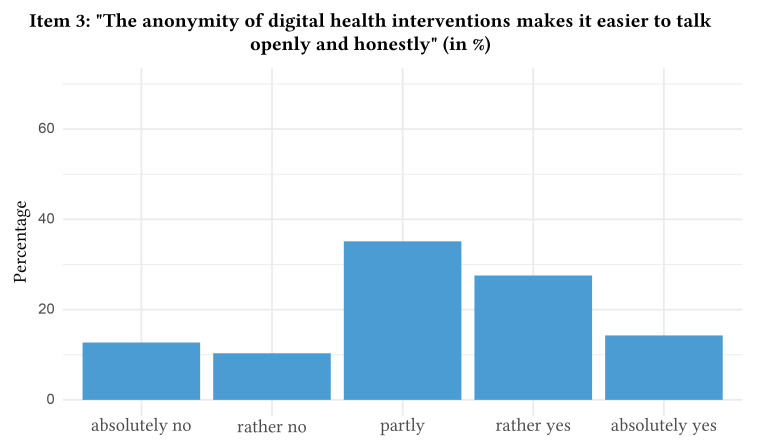
Attitudes Towards Digital Health Interventions: Item 3

**Figure 4:**
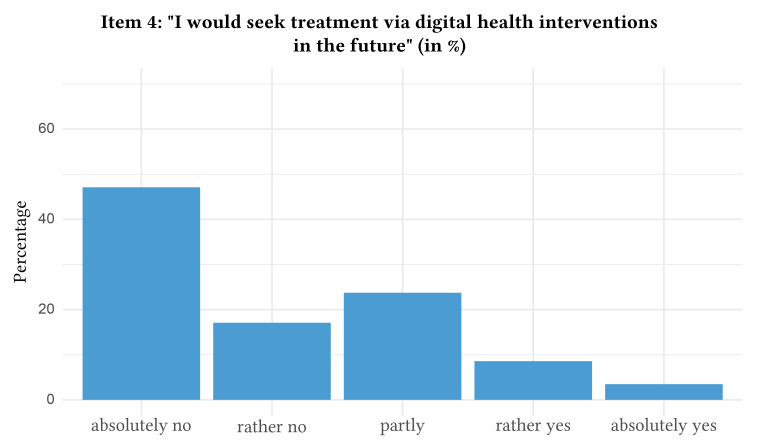
Attitudes Towards Digital Health Interventions: Item 4

**Figure 5:**
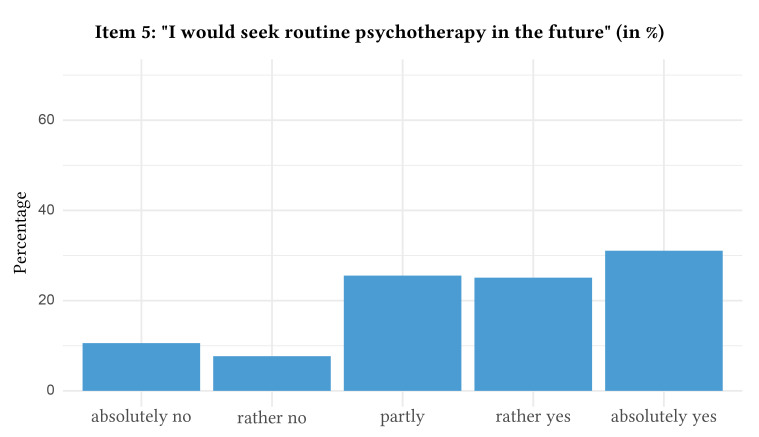
Attitudes Towards Routine Face-to-Face Psychotherapy: Item 5

The distribution of responses with regard to attitudes towards digital health interventions can be found in Figures 1, 2, 3, 4, and 5.

In detail, when asked if they would agree that they rated digital health interventions positively and as helpful, a majority of participants indicated partial agreement (i.e., rated Item 1 with 3 = partly, *n* = 1,046, 41.5%, 95% CI [39.6%, 43.4%]). Likewise, the statement that digital health interventions will reach and help more patients was approved partly by the majority of the participants (i.e., rated Item 2 with 3 = partly, *n* = 959, 38.1%, 95% CI [36.2%, 40.0%]). The majority also indicated partial agreement towards the statement that the anonymity of digital health interventions makes it easier to speak openly and honestly about important problems (i.e., rated Item 3 with 3 = partly, *n* = 854, 34.0%, 95% CI [32.1%, 35.8%]). Nevertheless, the majority of participants stated that they will “absolutely not” use digital health interventions for future psychological problems (i.e., rated Item 4 with 1 = absolutely no, *n* = 1,153, 45.8%, 95% CI [43.8%, 47.7%]). In contrast, the majority absolutely agreed that they would use routine face-to-face psychotherapy in case of future psychological problems (i.e., rated Item 5 with 5 = absolutely yes, *n* = 760, 30.2%, 95% CI [28.4%, 32.0%]). Of note, 10.2% of the participants (*n* = 256, 95% CI [9.0%, 11.3%]) rated Item 4 higher than Item 5, which indicates a preference for digital health interventions over routine face-to-face psychotherapy. The mean age of the subsample preferring digital health interventions was 42.8 (*SD* = 16.1).

### Correlational Analyses of Attitudes Towards Digital Health Interventions

There was no significant correlation between attitude towards digital health interventions and gender (i.e., sum score of Items 1-4; *r* = .002, *p* = .937) or current psychological stress (*r* = 0.000, *p* = .998). The correlation between age and attitude was moderate (*r* = -0.296, *p* < .001), with younger age being associated with higher acceptance of digital health interventions. The correlation between highest level of education and attitude towards digital health interventions showed a small positive association (rho = 0.14, *p* < .001). Responses to all individual items depending on gender and categorized by age groups can be found in the Supplementary Materials (see Steubl et al., 2025S).

### Open Questions

Out of the total number of participants, 17 (0.7%) stated that they have previously used a digital health intervention. Conditions mentioned included borderline personality disorder (*n* = 2; 11.8%), anxiety disorders (*n* = 2; 11.8%), depression (*n* = 2; 11.8%), obsessive-compulsive disorder (*n* = 2; 11.8%), addiction (*n* = 1; 5.9%), migraine (*n* = 1; 5.9%), burnout (*n* = 1; 5.9%), kleptomania (*n* = 1; 5.9%), and stroke (*n* = 1; 5.9%). The remaining six participants (35.3%) did not specify for which condition.

A total of 582 (23.1%) participants listed one or more conditions for which digital health interventions were considered as suitable treatment options. Most frequently listed were depression (*n* = 225, 38.7%) and anxiety disorders (*n* = 123, 21.1%). Other conditions mentioned were medical conditions (e.g., cancer, diabetes; *n* = 96, 16.5%), addictive disorders (*n* = 55, 9.5%), psychotic disorders (*n* = 15, 2.6%), obsessive-compulsive disorders (*n* = 13, 2.2%), eating disorders (*n* = 11, 1.9%), suicidal ideation (*n* = 8; 1.4%), personality disorders (*n* = 6, 1.0%), somatoform disorders (*n* = 4, 0.7%), and trauma-related disorders (*n* = 3, 0.5%). Additionally, 111 (19.1%) participants mentioned psychosocial problems not directly associated with medical or mental conditions.

Altogether, 822 participants (32.6%) stated possible barriers to initiating treatment via digital health interventions. Most commonly stated barriers were the absence of personal contact (e.g., no personalization, no therapeutic relationship, anonymous treatment, *n* = 285, 34.7%), technical aspects (e.g., no soft- and hardware, no Internet connection, problems with Internet in general, *n* = 164, 20.0%), and mistrust because of data privacy and security concerns (*n* = 124, 15.1%). Other mentioned barriers were not enough experience or competence in the use of such interventions or the Internet in general (*n* = 106, 12.9%), integrity of interventions (*n* = 98, 11.9%), costs of digital health interventions (*n* = 27, 3.3%), limited offer of digital health interventions (*n* = 23, 2.8%), limited perceived effectiveness (*n* = 23, 2.8%), health reasons (e.g., symptom severity, *n* = 7, 0.9%), and assessments that need to take place in-person (e.g., electrocardiogram; *n* = 3, 0.4%).

## Discussion

This representative study shows that attitudes towards digital health interventions in Germany are rather restrained. This holds particular significance because even the most effective digital interventions have limited impact if they are underutilized due to negative attitudes and lack of acceptance. Specifically, 45.8% of the representative sample in this study explicitly expressed an absolute reluctance to use digital health interventions for mental health in the future. In particular, older people and people with a lower level of education rated digital health interventions for mental health problems with reservation. This is in line with previous results suggesting that especially older adults experience a variety of barriers to the uptake of digital health interventions for mental conditions including the lack of trusted facilitators (Pywell et al., 2020; Wykes & Brown, 2016). However, even though there are concerns, there is still considerable acceptance in certain groups that also offers the opportunity to bring more individuals with mental conditions into treatment. For example, when looking at the youngest age group (< 20 years) this percentage drops to 27.3% with the majority (28.4%) stating that they would partly seek treatment via digital health interventions in the future (for details see Supplementary Materials [Steubl et al., 2025S]). Additionally, there is also a subsample (*n* = 256, 10.2%) that seems to prefer digital health interventions over routine face-to-face psychotherapy and a considerable number of participants (*n* = 595, 23.6%) listed mental health conditions they could see digital health interventions being used for. Accordingly, the frequently expressed barriers assessed within the survey (i.e., absence of face-to-face therapeutic relationship with personalization, technical aspects, and data privacy and security concerns) are of particular importance.

Hereby, the primary concern lies in the absence of a genuine therapeutic relationship and the perception that digital health interventions are unable to provide the same level of personalized treatment as traditional face-to-face psychotherapy. However, it is well established that it is indeed possible to form a therapeutic relationship in (guided) digital health interventions (Berger, 2017; Flückiger et al., 2018; Pihlaja et al., 2018). Additionally, emerging viewpoints suggest that while a stable therapeutic relationship is of importance in conventional treatment, digital health interventions may extend beyond this and be applied for alternative purposes (e.g., micro interventions or to support significant others; Baumel et al., 2020; Baumel & Tamir, 2022). These novel applications appear to diminish the significance of the therapeutic relationship, as perceived by patients (Baumel & Tamir, 2022). Moreover, there is a variety of new approaches to make digital health interventions more personalized and engaging with possible approaches include just-in-time adaptive interventions, gamification, chatbots, and interventions that are personalized and tailored to the individual (Baumeister et al., 2023; Baumeister & Montag, 2023; Domhardt, Cuijpers, et al., 2021; Torous et al., 2021). While the issue of missing technical prerequisites (e.g., an Internet connection) cannot be solved that easily, the ubiquitous digital change may lessen this issue in the future.

Finally, privacy and security concerns are certainly to be taken very seriously as digital health interventions are dealing with private and sensitive data. However, this problem may be of special relevance when using digital health interventions from unknown sources. Fortunately, there is a wide range of digital health interventions from reputable and well-established sources with a high level of data protection efforts on the market. Additionally, there are platforms available online that rate interventions with regard to for example data privacy and security details (e.g., Mobile Health App Database, mhad.science [Stach et al., 2020] or https://onemindpsyberguide.org/). Moreover, patients in Germany have the opportunity to use so-called DiGAs. These digital interventions are subject to certain data privacy and security regulations to protect users' personal health information (Ludewig et al., 2021). Patients can obtain DiGAs with a prescription by their consulting physician, medical doctor or psychotherapist and use them as part of their treatment plan (Ludewig et al., 2021). Nevertheless, the exemplary regulation of digital interventions in Germany and other countries like Sweden or Australia does not refer to all available digital health interventions and many reviews of publicly available mobile health apps show considerable limitations when it comes to data privacy and security (Domhardt, Messner, et al., 2021; Simon et al., 2023; Steubl et al., 2022).

Taken together, the barriers seem to reflect low scores on the UTAUT dimensions *Facilitating Conditions* and *Effort Expectancy*. Interestingly, only a minority of participants explicitly questioned the effectiveness of digital mental interventions (2.8%) and none mentioned the influence or opinions of others as a reason for non-use (0.0%), despite *Performance Expectancy* and *Social Influence* being core factors within the UTAUT model. While the emphasis on the absence of personal contact could also reflect a broader skepticism regarding the performance of digital interventions, and thus relate to *Performance Expectancy*, these findings suggest that efforts to increase acceptance and uptake may benefit from a focus on improving perceived usability, trust, and the integration of relational elements, rather than solely emphasizing efficacy.

Of note, the majority of participants (67.4%) did not provide any response when asked about potential barriers to using digital health interventions. This may reflect a lack of specific concerns among many respondents, but it could also be due to the open-ended nature of the question, which typically yields lower response rates compared to closed questions with multiple choice answers provided. Additionally, participants may have lacked detailed knowledge about such interventions, which could limit their ability to anticipate barriers.

Nevertheless, it seems evident from the aforementioned points that there exist valid reasons for patients to hold a more positive stance towards reputable digital health interventions than it is currently the case. Fortunately, there are numerous ways to influence and develop patients’ attitudes positively (Bauer & Kirchner, 2020). For example, as a persuasive strategy, proponents have advocated the incorporation of future users and individuals with lived-experience in the developmental and implementation phases of digital health interventions (i.e., participatory development; Geirhos et al., 2021; Hochmuth et al., 2020). Moreover, there have been successful efforts to offer acceptance-facilitating interventions to both patients and therapists (Baumeister et al., 2014; Ebert et al., 2015). In general, it seems likely that a (corrective) experience with a digital intervention or more knowledge on the matter (e.g., through educational campaigns) is necessary to alleviate concerns or apprehensions. This should be addressed in depth in future studies and efforts.

In addition to promoting acceptance of digital health interventions, our findings may also inform future strategies on how to implement these interventions within the healthcare system. Specifically, the results highlight the importance of offering a variety of treatment formats (e.g., face-to-face/on-site, video-based/remotely delivered, fully digital/stand-alone) in routine mental health care. Given that individual preferences – such as the desire for personal contact – can shape attitudes and acceptance, providing freedom of choice and for example blended therapy formats (Baumeister et al., 2018) may help align treatment settings with patients’ expectations and increase engagement.

It is worth highlighting that in contrast to attitudes toward digital interventions, a substantially larger proportion of participants indicated that they would be willing to use routine face-to-face psychotherapy in the case of future psychological problems. These results reflect the alignment between public attitudes and the current organization of the mental health care system, in which face-to-face psychotherapy remains the standard form of service delivery next to (rudimentary) mental healthcare provided by general practitioners (e.g., with medication). It also shows greater familiarity and comfort with traditional treatment modalities, as well as a general preference for direct personal contact, which was also a commonly cited barrier to using digital intervention formats. These results further underscore the continued importance of offering and extending face-to-face treatment options – while addressing some limitations of on-site treatments like long waiting times at the same time – and highlight that digital health interventions may complement, rather than replace, conventional care in mental health services (e.g., as in blended therapy formats; Baumeister et al., 2018) and may represent a low-threshold component in stepped mental healthcare (Domhardt & Baumeister, 2018).

### Limitations

While the results of this study shed light on the acceptance of digital health interventions, there are some important limitations to consider. First and foremost, the term digital health intervention is very broad and participants were only provided with a concise description comprising two sentences. In the following, some participants might have had a very limited or unrealistic understanding of the concept resulting in differences in attitudes. Additionally, blended therapy approaches (i.e., the combination digital health interventions with traditional face-to-face therapy) were not explicitly mentioned in the survey. Given that such formats are generally associated with higher acceptance compared to stand-alone digital interventions (Schuster et al., 2018), the incorporation of this treatment format into the survey might have had an additional influence on participants’ responses. While the decision not to prime participants with specific interventions may have certain advantages (e.g., not focusing on one certain intervention), it might be beneficial for future studies to actually present participants with one or two examples (similar to the aforementioned acceptance-facilitating studies; Baumeister et al., 2014; Ebert et al., 2015). This is especially important, as a considerable amount of the replies to the open questions might have focused on video-based therapy. While this is certainly an important and already widely used form of a digital intervention as outlined previously in this paper, it might not provide the same advantages (e.g., cost-effectiveness, scalability) and disadvantages (e.g., limited guidance, concerns regarding therapeutic relationship) as other digital health interventions (i.e., IMIs) and might have to be viewed separately (for a more detailed discussion on this issue: Steubl & Baumeister, 2023). Second, the reported statistical analyses provide predominantly a descriptive picture of the gathered data. Longitudinal designs and examining changes over time would be highly worthwhile. Third, potentially relevant individual characteristics were not assessed (e.g., digital literacy, stigmatization of mental conditions, ethnic background) due to the broader scope of the overall study of which our survey represents a specific sub-analysis. As a result, our ability to examine how these factors may influence attitudes towards digital interventions is limited, and this may affect the generalizability of our findings to all segments of the population. Last, this study assesses attitudes towards digital health interventions, but considering the use of certain offers in a questionnaire might be different to actually using them. However, the UTAUT model on which the employed questionnaires are based strongly suggests that assessed acceptance is a direct predictor of use of digital offers (Venkatesh et al., 2003).

### Conclusion

This study suggests that a majority of adults in Germany would (rather) not use digital health interventions. However, this reservedness decreases with younger age and there is a subsample of individuals who would rather use digital health interventions than regular face-to-face treatment. The identified barriers for the usage of digital interventions (i.e., absence of personal contact, technical issues, and concerns related to data privacy and security) need to be addressed in order to fully harness the potential of digital health interventions in routine care.

## Supplementary Materials

The Supplementary Materials contain the following items (for access, see Steubl et al., 2025S):

- Table 1. Full questionnaire (translated)
- Figure 1. Attitudes towards digital health interventions depending on gender
- Figure 2. Attitudes towards routine face-to-face psychotherapy depending on gender
- Figure 3. Attitudes towards digital health interventions depending on age group
- Figure 4. Attitudes towards routine face-to-face psychotherapy depending on age group

SteublL. S.
BüscherR.
SanderL. B.
BaumelA.
BarckK.
SachserC.
FegertJ. M.
BrählerE.
BaumeisterH.
DomhardtM.
 (2025S). Supplementary materials to "Attitudes towards digital health interventions in Germany: Findings from a population-based representative survey"
[Full questionnaire and additional figures]. PsychOpen. 10.23668/psycharchives.18889
PMC1292319141727813

## Data Availability

Data, code, and materials can be obtained by contacting the first author upon reasonable request.

## Social Media Accounts

*Lena Sophia Steubl:* LinkedIn.

## Preregistration

The present study analyzes selected data from a large, population-based representative survey conducted in Germany. As the analyses were conducted retrospectively based on an existing dataset, the study was not preregistered.

## Reporting Guidelines

The paper follows the JARS–Quant guidelines for quantitative research.
